# Brain activity underlying visual search in depth when viewing volumetric multiplanar images

**DOI:** 10.1038/s41598-023-34758-9

**Published:** 2023-05-11

**Authors:** Mehrdad Naderi, Tatjana Pladere, Reinis Alksnis, Gunta Krumina

**Affiliations:** 1 Department of Optometry and Vision Science, Faculty of Physics, Mathematics and Optometry, University of Latvia, Riga, Latvia; 2 Laboratory of Statistical Research and Data Analysis, Faculty of Physics, Mathematics and Optometry, University of Latvia, Riga, Latvia

**Keywords:** Cognitive neuroscience, Visual system, Neuroscience

## Abstract

The study investigated the cortical activity associated with 3D and 2D image perception on a volumetric multiplanar display by analyzing event-related potentials (ERPs) and power spectral density (PSD). In this study, we used a volumetric multiplanar display to present visual targets, and the brain signals were recorded via an EEG amplifier and analyzed using the EEGLAB toolbox on MATLAB. The study found no significant differences in amplitude between the 3D and 2D conditions across five occipital and parietal electrodes. However, there was a significant difference in latency of the P3 component on the Pz electrode. The analysis of PSD showed no significant differences between the two conditions, although there was a slightly higher alpha and beta activity observed in the 2D visualization. The study concluded that 3D image representation on a volumetric multiplanar display has no more sensory or cognitive load on the human brain than 2D representation, and that depth perception on a multiplanar display requires less brain activity.

## Introduction

By developing the three-dimensional (3D) technologies, the demand for high-quality images keeps growing, resulting in the development of various 3D presenter tools in both head-mounted and front-view displays^1,2^. In the field of human–computer interaction, the crucial question is whether the depth effect produced by the new method brings benefits to users to perceive spatial relationships among displayed objects. Answering this question depends on the physical properties of the generated visual stimuli related to the display technology and the specifics of human visual perception^3^. Therefore, assessing the ergonomics of 3D presenter tools has become essential in terms of depth perception and deployment of visual attention^4,5^. Visual search in 3D environment depends on depth perception^6,7^ because it relies on our ability to selectively attend to certain features or objects in a visual scene and perceiving them, moreover, the depth cues have an essential role in depth judgment, and applying more depth cues results in more direct depth perception^8,9^.

Depth cues are sources of information on the weight of changes depending on the viewing condition^10^. At close viewing distances, the relative binocular disparity is considered a prerequisite for accurate depth perception^10,11^. Visual search in 3D visualization depends on depth perception^6,7,12^ because it relies on our ability to selectively attend to certain features or objects in a visual scene and perceiving them, moreover, classical models of visual search^13,14^ propose that attention plays an active role in both early and late visual processing. Studies have found that the brain processes information about depth, which is available early in visual processing and becomes more prominent in higher-order representations. Specifically, differences in brain activity between viewing stereoscopic and two-dimensional images have been observed in parietal and occipital regions at 90–130 ms after visual stimulus onset^15^ used functional magnetic resonance imaging (fMRI) to examine stereo processing in V1 and other areas of visual cortex^16^, studied evoked potential components that are generated exclusively by cortical structures when dynamic random-dot stereograms (dRDS) were presented^17^, explored cortical responses to dichotically presented random-dot (RD) stimuli which formed a checkerboard by means of horizontal disparity by using fMRI^18^, studied slow cortical potentials and source localization to identify the neural correlates of monocular and binocular depth cues^19^, studied neurophysiological correlates of vertical disparity in 3D images using EEG and specifically P1 component of ERP, and^20^ concluded real-world objects trigger stronger and more sustained action-related brain responses than images do. This highlights the important role of these brain areas on processing depth information. Some studies have reported slightly earlier^21^ or later^22,23^ reactions to depth perception in the brain.

In addition to neural indicators of early sensitivity to depth, highlighted differences in the later cognitive processes^7,23–25^ showing that amplitudes correlated with the absolute values of binocular disparities^24^ and orienting of attention^26^ at 150–200 ms. In later times, the stereoscopic input modulated high-level perceptual processes involved in the integration of information^24–27^, figure-ground segmentation^7,23,25^, view generalization^21^, and recognition^19^. These processes were primarily associated with neural activity in the parietal region rather than the occipital region.

Most of the research centered on clarifying the neural activity correlates to the binocular disparity processing that was performed using stereoscopic images ranging from anaglyph-based^21,23,27^ to polarization-based^19,28^, however, to the best of our knowledge, there are no studies about the actual depth perception and the direct brain-behavior of actual depth perception. Stereoscopy creates the illusion of image depth by separating visual inputs for both eyes, thus possibly causing binocular vision stress due to the accommodation-vergence conflict that can induce discomfort and visual fatigue^8,28–32^. On the other hand, actual depth is free of the mentioned issues since no conflict between accommodation and vergence is present. Electroencephalography (EEG) is a reliable method because of its high temporal resolution in order to measure the interaction of human ergonomic properties with physical factors, especially in the field of visual system evaluation for instance^29,31,33^, studied on mental and visual fatigue caused by 3D TV^30^, Quality of experience of stereoscopic 3D TV^28,32^, discomfort evaluation and visual comfort estimation during stereoscopic visualization. Compared to behavioral measures, the neurophysiological one is less biased^28^.

New approaches are being developed to avoid the forceful separation of views for both eyes to improve user comfort and performance. The new displays aim to provide three-dimensional images on multiple focal planes, that it requires no polarized or anaglyph eyewear^1,8,34–36^. Specifically, image points in the physical space of optical elements can be shown in a time-multiplexed manner^1,35,37^.

Depth perception plays an essential role in visual search when finding objects in space or information in spatial images. It is crucial both in our daily lives and in performing professional tasks that involve qualitative or quantitative judgments about the third dimension of images. As new three-dimensional systems such as virtual reality, augmented reality, holographic 3D displays, and volumetric multiplanar displays are intended to be used daily, it is crucial to understand how their implementation affects user performance for instance, error rate, response time, efficiency, and satisfaction. Previous behavioral studies provided some experimental support for using volumetric multiplanar display instead of stereoscopic images by reporting more accurate judgments on spatial relationships^47^ and faster information recognition time^34^. However, to our knowledge, no studies have yet assessed how neural activity changes in response to new 3D displays.

The present exploratory study aimed to assess the EEG features of an actual 3D visual search. We hypothesized that it could cause lower amplitude in ERPs and Power Spectral Densities (PSD) to find the closest visual element when presented in the form of 3D volumetric images compared to the state that no depth difference exists between elements. So far, EEG was broadly employed to study visual search^25,26^ and depth perception^15,18,23,38^ for stereoscopic visualization, however, the application of these findings is limited for the understanding of information processing for viewing 3D volumetric images. In the current EEG study, an experiment was performed to investigate the electrophysiological indicators of differences depending on the 3D and 2D volumetric multiplanar image as a real 3D perception.

## Materials and methods

In this study, we investigated brain activity during a visual search when individuals viewed volumetric images and answered a visual question. Two different image presentations, i.e., 3D and 2D visual stimuli, were applied to compare information processing requirements. The study was approved by the Ethics Committee of the University of Latvia (ZD2019/20807) and was conducted following the Declaration of Helsinki.

### Participants

Twenty participants (9 males, 11 females) with a mean age of 25 ± 6 years voluntarily participated in the study. We assessed the optometric visual tests before the experiment to ensure their binocular vision function. The participants' inclusion criteria were the following: normal or corrected-to-normal visual acuity (1.0 or better, decimal units), no binocular dysfunctions, stereoscopic acuity of 40 arcsec or better (assessed using a Titmus stereo test, Stereo Optical Co., Chicago, IL). At the end four participants excluded from the experiment. Two of them are due to failing the optometric criteria and the rest due to noisy brain signals and high rate of mistake in task performance. The remained participants were 16 subjects (6 males, 10 females) with a mean age of 25 ± 6 years. All participants were unaware of the specific purpose of the study.

### Apparatus

The solid-state volumetric display (LightSpace Technologies, model: X1406, 19" diagonal) was used to show the visual stimuli. The display contains twenty physical image depth planes and short liquid–crystal-based light diffuser elements acting as temporary image receiving screens when synchronously coupled to a high refresh-rate image projection unit^35^. In operation, diffusers are switched between the highly transparent and light diffusing state at a high rate, ensuring an overall volumetric image refresh rate of 60 Hz. The resolution of the displays is 1024 × 768 pixels per image depth plane.

### Procedure and Task

First, the procedure and the aims of the task were explained to the participants. Then, the participants provided written informed consent. Participants' visual acuity and stereoscopic acuity were screened. The experiment was conducted in a scotopic (0.1 lx) condition.

**Figure 1:**
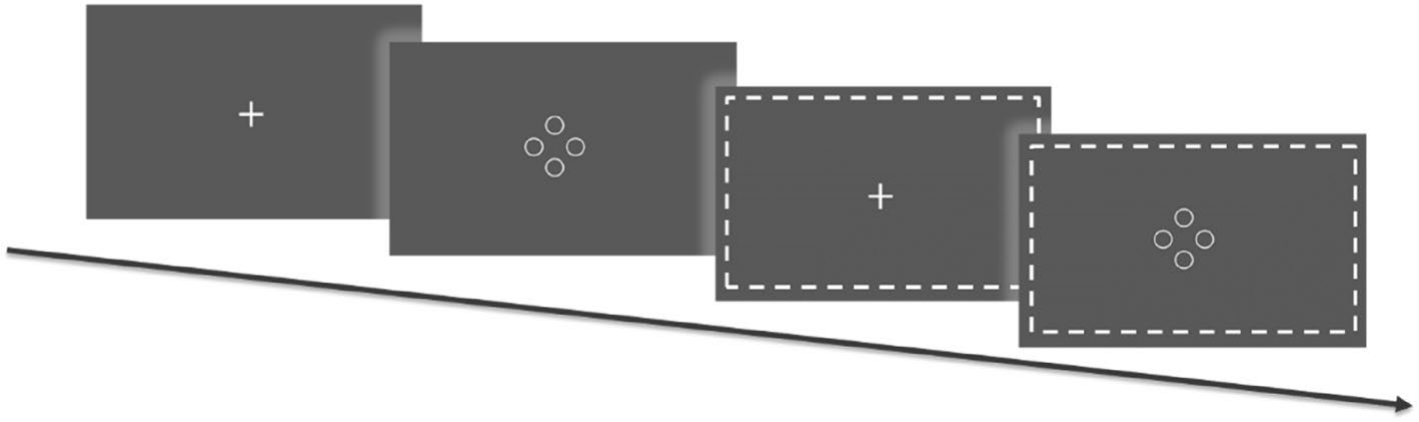
The experiment design paradigm contained 160 trials of ring presentation that half trials had a different binocular disparity.

**Figure 2:**
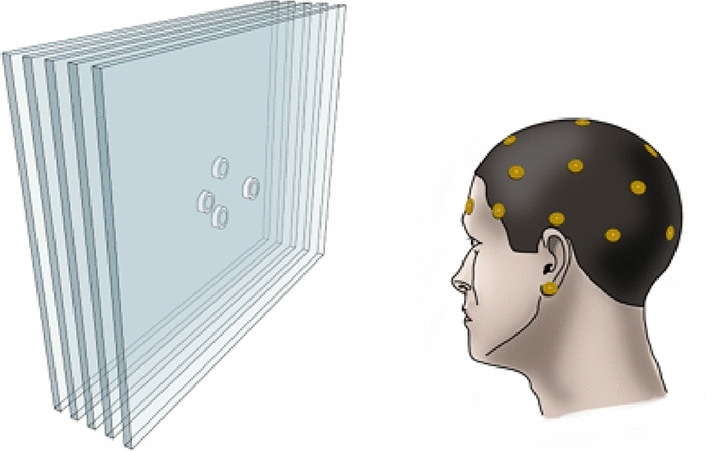
Schematic illustration of the setup and stimulus in a 3D trial. In the search array, a target was presented per plane closer to the participant compared to the distractors on the multi-plane display.

The whole experiment included a total of 160 trials. The 3D demonstration occurred in 50% of the trials in a pseudo-randomized order. Each trial started with a fixation cross that was presented in the middle of the screen for 1 s. Next, four circles (outer diameter – 0.5°, line width – 0.1°) were displayed at 1.0° field eccentricity from the display center. In the 3D trials, one circle (a target) had a different binocular disparity in comparison to three others (distractors), because of which it appeared closer to the viewer, shown in Figs. 1 and 2. Participants had to search for a target and report its relative location within the display by choosing one of four responses (up, right, down, and left). For the response, the arrow keys of a computer keyboard were used. After the response submission, the fixation cross reappeared, and the subsequent trial followed. The entire time of the task performance was on average 10 min depending on the response time. All target positions were counterbalanced across directions. In the 2D trials, all circles were presented at one depth plane; thus, the search elements contained equal disparity. The subjects responded to the 2D trials by pressing the space button on the keyboard. The experiment was not time constrained. However, the participants were instructed to complete the task as accurately and quickly as possible. Participants sat facing the display at a viewing distance of 90 cm (see Fig. 2).

### EEG data acquisition and analysis

The brain's electrical activity was captured with a Nicolet v32 EEG system. Twenty-one active electrodes were positioned following the international 10–20 system^39^, and the average of all active electrodes was chosen as a reference. A sampling rate of 1024 Hz was selected for data collection, and a band-pass filter was applied from 1 to 70 Hz. Data were recorded continuously during the visual search tasks whilst electrode impedance was kept below 10 KΩ. Data were saved alongside triggers marking significant events. EEG data analysis was performed using the open-source toolbox EEGLAB 2019.1.0 connected to MATLAB R2018a (MathWorks Inc., Natick, MA, USA).

The EEG recordings for every participant were purified by the noise rejection, for example, removing baseline, blinking, and muscle activity based on a two-step procedure that included the built-in software algorithm and visual inspection of variance. The signals were separated into time-locked epochs of 1200 ms duration synchronized with the onset of search arrays, containing 200 ms of prestimulus. For everyone, the epochs were averaged according to the stimulus condition (3D and 2D trials). Only correct responses were analyzed, excluding trials with false alarms or misses. Three separate time windows were selected corresponding to different cognitive processes in visual search and depth perception^18,19,24,25,40^ to study the EEG signal variation at the temporal level. Thus, the amplitudes were calculated as the mean potentials in the following time windows: 50–100 ms, 100–200 ms, and 200–450 ms. Correspond to components N1, P2, and P3, respectively. The amplitudes of ERPs on five electrodes positioned over the parietal and occipital regions (O1, O2, P3, P4, and Pz) were investigated in detail. Moreover, the Power Spectral Density (PSD) of alpha and beta waves were analyzed to assess the cognitive properties of the volumetric multiplanar image depth perception.

### Statistical data analysis

At first, to analyze the ERP data, the given time interval was divided into three subintervals: (1) 50–100 ms; (2) 100–200 ms; (3) 200–450 ms. Then the smallest response value for each subject was determined within the first interval, and the most considerable value was determined within the second and third intervals. The distributions of the obtained values were relatively symmetric with no apparent outliers; hence the paired t-test was used to test for significant differences between the two conditions (for each electrode and subinterval). In total, 15 tests were performed. Hence some adjustment methods such as Bonferroni or Holm must be applied when the results are interpreted. However, in the paper, original p-values are reported. In the analysis of frequency band data, the paired Wilcoxon signed-rank test was used to test for significant differences between the two conditions at each of the given frequencies for each electrode. Although for most of the frequency, normality could not be rejected, and paired t-tests could be used, which is known to have a more extensive power in detecting significant effects.

## Results

### Performance Data

The behavioral results indicated that across all participants, the mean correct response rates for 3D stimuli were 0.98 (SD = 0.04); however, more errors were made when individuals responded to 2D stimuli on the volumetric display. Specifically, the correct response rates dropped to 0.81 (SD = 0.06) when the 2D stimuli were shown.

**Figure 3:**
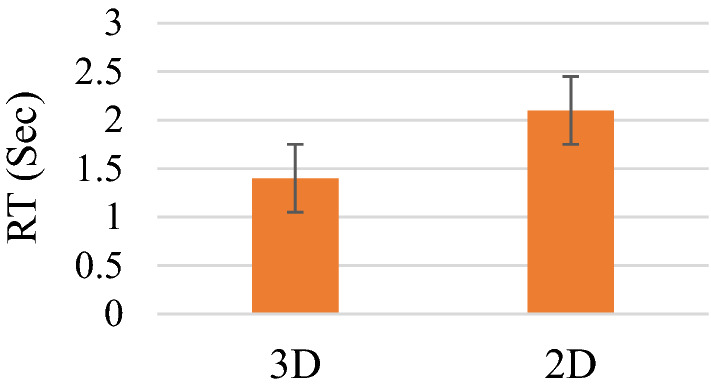
Averaged response times when the visual stimuli were shown as 2D and 3D. Error bars depict standard deviations.

In addition to the correct response rates, response times (RT) were analyzed to evaluate visual search outcomes. The statistical analysis revealed in Fig. 3 that there were no significant main effects of the stimulus condition.

### Event-related potentials (ERPs)

**Figure 4:**
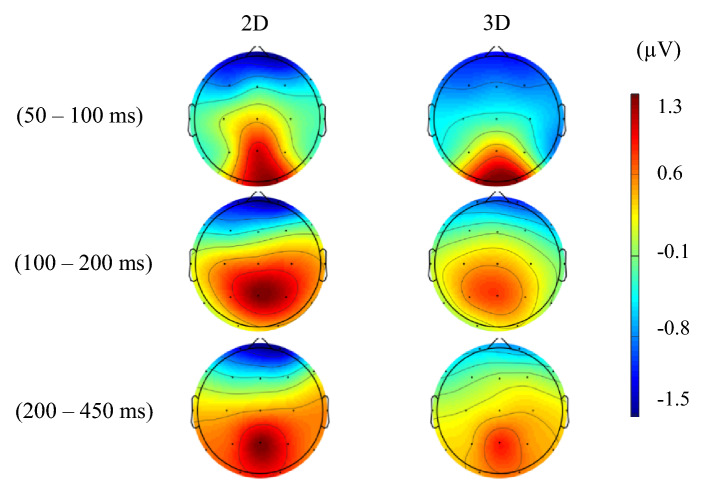
ERP topographical maps reflecting the brain activity during three time windows, averaged across all participants when performing the 2D task and 3D task on volumetric multiplanar display.

**Figure 5:**
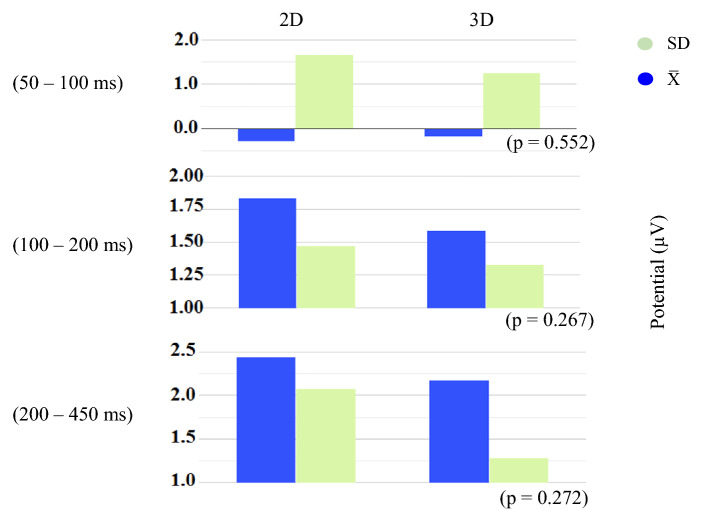
Average and standard deviation of five electrodes P3, P4, O1, O2, and Pz across all participants. P-value represents no statistically significant differences in each time window.

We analyzed the cortical signals in three time windows corresponding to ERPs' N1, P2, and P3 components. Generally, higher activation of occipital and parietal was seen as expected. Figure 4 shows changes in amplitudes of ERPs at three time windows averaged over all sixteen participants in the form of topographical maps. Moreover, Fig. 5 shows the same time windows in the form of bar chart including information about the standard deviation and p-value.

### Waveforms

**Figure 6:**
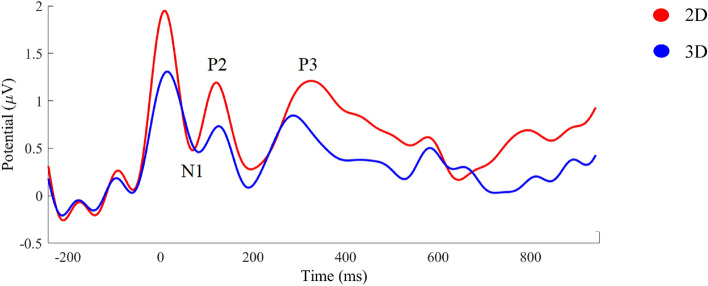
The waveform average of five electrodes O1, O2, P3, P4, and Pz in two conditions between − 200 to 1000 ms, across 16 subjects. Statistical analysis showed no significant differences between the two conditions.

P1 component is not easily detectable; however, it occurs approximately after 50 ms of stimuli onset^41^. Waveform showed the P1 component was presented as a negative deflection, therefore we had N1 component. Since the ERPs components are dominant waves on the occipital and parietal areas, the statistical analysis applied on five electrodes (O1, O2, P3, P4, and Pz). Figure 6 indicates the properties of the waveform results. Since we had N1 component, we considered the minimum point of each waveform to perform the statistical analysis. Moreover, we chose the max value of P2 and P3 components. The results showed there was no significant differences between two conditions in three time-windows across 16 subjects and for each electrode location.

The latency analysis of ERP components showed no statistically significant differences between the two conditions except for the Pz electrode and within the time-window 200–450 ms, which corresponds to the P3 component. The average latency results are summarized and reported in Table 1.

**Table 1:** The average latency of N1, P2, and P3 components over five electrodes in two conditions.

Electrodes	50–100 ms			100–200 ms			200–450 ms		
	2D	3D	*p* value	2D	3D	*p* value	2D	3D	*p* value
P3	75.5 ± 18	77 ± 16	0.217	155.5 ± 26	152.5 ± 24	0.701	299 ± 71	309 ± 73	0.677
P4	84 ± 13	85 ± 12	0.796	144 ± 22	138 ± 22	0.421	311 ± 84	322 ± 74	0.711
O1	87 ± 14	85.5 ± 18	0.848	141 ± 27	142 ± 27	0.92	320 ± 70	336 ± 65	0.524
O2	88 ± 11	83 ± 17	0.284	132 ± 22	142 ± 23	0.189	340 ± 55	338 ± 70	0.943
Pz	79 ± 17	73.5 ± 18	0.338	150 ± 27	140 ± 27	0.287	329 ± 68	279 ± 59	0.048

### Power spectral density (PSD)

**Figure 7:**
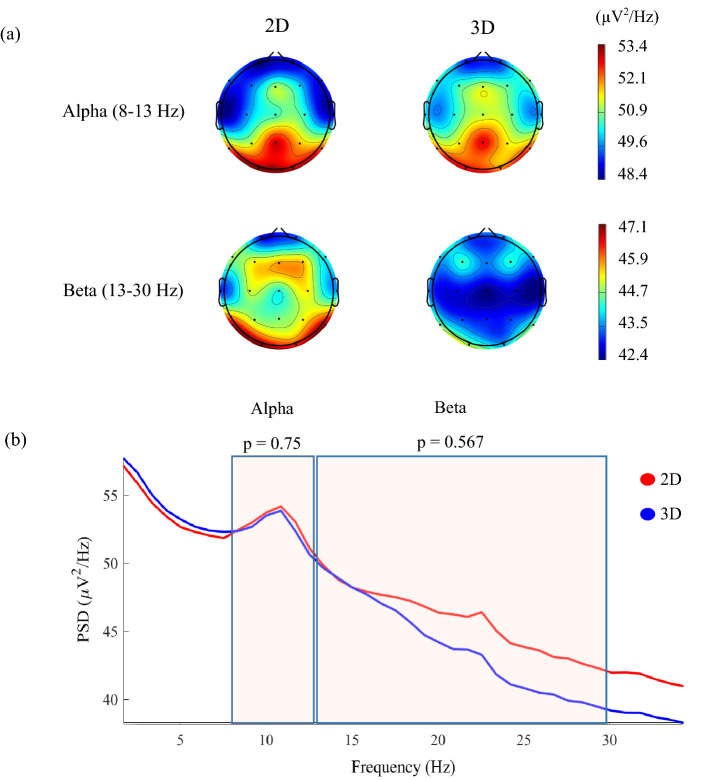
(**a**) Power Spectral Density (PSD) analysis in the form of the topographical map over the skull average of all participants. (**b**) The waveform of Alpha and Beta power. Beta wave shows higher activation in the 2D condition compared to the 3D.

Continuous wave analysis showed slightly higher activation in alpha and beta frequency bands; however, the difference was not statistically significant. Topographical maps and waveforms of alpha and beta showed in Fig. 7a and b, respectively.

### Ethical approval

The study was approved by the Ethics Committee of the University of Latvia (ZD2019/20,807) and was conducted following the Declaration of Helsinki.

## Discussion

In this study, we analyzed cortical activity in the human brain by analyzing ERPs and PSD over occipital and parietal electrodes under two conditions, 2D and 3D perception of volumetric visual stimuli representation. The goal was to determine if there were differences in amplitude and latency of ERP components as well as the amplitude of PSD regarding alpha and beta bands under two viewing conditions. Since this study focused on volumetric display, the results could indicate positive aspects of the interaction of the volumetric display with the human visual system, as there is no accommodation-convergence conflict in this examination.

Behavioral data is a logical way to prove or reject a hypothesis. Previous studies show that response time can be used as a factor for visual target perception^11,48^. In this study, behavioral data, which indicated shorter response time in the 3D task, could serve as logical proof of easier perception of 3D images on the volumetric display compared to 2D images, as reported in^34^. Since this study was time-free, the subjects had sufficient time and motivation to perform the task correctly.

The effect of the third dimension of the image was already observed in the brain activity associated with sensory resources and early information processing in the occipital and parietal regions at 50–100 ms after stimulus onset. However, its size was small for both visual targets, possibly due to similar physical properties and dimensions of 3D and 2D visual stimuli. Moreover, a slight difference could reflect involvement of early neural activity by receiving feedback from higher-level processing locations^16–23,27^. Interestingly, early perceptual sensitivity of ERPs to depth was reported not only for digital images [16, 19. 21–23, 27] but also for actual objects^20^. Furthermore, early cognitive responses did not vary depending on the type of visual target^27^ and the availability of consistent depth cues^28^. These findings suggest that early information processing can be invariant in response to within-dimension features.

The second time window (100–200 ms) is associated with cognitive processes such as working memory, memory performance, and semantic processing^42,43^. Although there was no statistically significant difference between the two types of images, when looking at topographical maps and waveforms, slightly higher activity can be seen in 2D image perception compared to 3D. This could be because presentation of both visual targets in a sequence of trials caused involvement of memory to differentiate the 2D visual target from the 3D one. Moreover, the P2 peak latency result showed no statistical differences between two conditions over five occipital and parietal electrodes across all subjects. This similar latency represents equal attention and memory function involved in visual data analysis by the visual cortex.

The P3 component is a dominant wave on the parietal cortical area that is typically observed after 250 ms of stimuli onset and is associated with decision making. The latency of this wave is influenced by the difficulty of decision making^44–46^. In our study, we observed a slightly higher amplitude of P3 associated with 2D perception, although this difference was not statistically significant. However, there was a significant difference in P3 latency between the 2D and 3D conditions over the Pz electrode. This finding suggests that discriminating 2D stimuli on a volumetric display may be more challenging for subjects performing 3D tasks. Interestingly, our results contradict those of^28^, who reported higher P3 amplitude in the comfort zone of virtual depth perception compared to the non-comfort zone.

Power Spectral Density (PSD) is a measure of the distribution of signal power over frequency, which can provide insight into how the brain reacts to stimuli. For example, higher activity in the alpha band is typically associated with lower attention, while higher activity in the beta band indicates a higher cognitive or problem-solving load on the brain^2^. Our analysis of PSD revealed that alpha and beta had slightly higher amplitudes (though not significant) during the 2D task, which may be due to additional processing demands required to discriminate 3D from 2D trials in the 2D experiment. Additionally, we examined parameters such as total power, spectral band power, and median and spectral edge frequency, which can provide further information about brain activity in response to stimuli.

## Conclusion

In this experiment, we studied the effect of the 2D and the 3D volumetric multiplanar image on human brain signals by analyzing EEG data. Event-related potentials in three time windows corresponding to N1, P2, and P3 components were analyzed, as well as the power spectral density of two frequency bands. In this work, generally, no significant differences over three time windows and alpha and beta frequency bands in two conditions were in line with our hypothesis that 3D perception on the volumetric multiplanar display has no extra load on human brain processing.

## Data Availability

The analyzed data of ERP and frequency bands are available in google drive, https://drive.google.com/drive/folders/1ZjsXrSywL-1LCm6VC2YOU71KB-twfJhf. The statistic analysis is available from the corresponding author on reasonable request.

## References

[1] Geng J (2013). Three-dimensional display technologies. J. Adv. Opt. Photon..

[2] Naderi M, Pladere T, Krumina G (2020). EEG based assessment of user performance for a volumetric multi-planar display. J. Digit. Opt. Immers. Displays.

[3] Jacko. *Human Computer Interaction Handbook: Fundamentals, Evolving Technologies, and Emerging Applications* (ed. Julie A. Jacko) 157–192 (CRC Press, 2012).

[4] Huynh-Thu Q, Barkowsky M, Le Callet P (2011). The importance of visual attention in improving the 3D-TV viewing experience: Overview and new perspectives. J. IEEE Trans. Broadcast...

[5] Poulakos S, Roethlin G, Schwaninger A, Smolic A, Gross M (2014). Alternating attention in continuous stereoscopic depth. ACM.

[6] O’Toole AJ, Walker CL (1997). On the preattentive accessibility of stereoscopic disparity: Evidence from visual search. J. Percept. Psychophys..

[7] Finlayson NJ, Remington RW, Retell JD, Grove PM (2013). Segmentation by depth does not always facilitate visual search. J. Vision..

[8] Hoffman DM, Girshick AR, Akeley K, Banks MS (2010). Vergence-accommodation conflicts hinder visual performance and cause visual fatigue. J. Vision..

[9] Reichelt, S., Haüssler, R., Fütterer, G., & Leister, N. Depth cues in human visual perception and their realization in 3D displays. *J. Three-Dimensional Imaging, Visualization, and Display 2010 and Display Technologies and Applications for Defense, Security, and Avionics IV*. **7690**, 92–103 (2010).

[10] Howard, I. P., & Rogers, B. I. *Perceiving in Depth: Stereoscopic Vision* (ed. Ian P. Howard, Brian J. Rogers) 385–386 (Oxford University Press, 2012).

[11] Rogers B (2019). Toward a new theory of stereopsis: A critique of Vishwanath. J. Psychol. Rev..

[12] Plewan T, Rinkenauer G (2019). Allocation of attention in 3D space is adaptively modulated by relative position of target and distractor stimuli. J. Attent. Percept. Psychophys..

[13] Treisman A, Gelade G (1980). A feature-integration theory of attention. J. Cogn. Psychol..

[14] Wolfe, J. M. *Integrated Models of Cognitive Systems* (ed Gray, W. D) 99–119 (Oxford University Press 2007).

[15] Backus BT, Fleet DJ, Parker J, Heeger DJ (2001). Human cortical activity correlates with stereoscopic depth perception. J. Neurophysiol..

[16] Skrandies W (2001). The processing of stereoscopic information in human visual cortex: Psychophysical and electrophysiological evidence. J. Clin. Electroencephalography..

[17] Rutschmann RM, Greenlee MW (2004). BOLD response in dorsal areas varies with relative disparity level. J. Neuroport..

[18] Fischmeister FPS, Bauer H (2006). Neural correlates of monocular and binocular depth cues based on natural images: A LORETA analysis. J. Vis. Res..

[19] Avarand FS (2017). Objective quality assessment of stereoscopic images with vertical disparity using EEG. J. Neural Eng..

[20] Marini F, Breeding KA, Snow JC (2019). Distinct visuo-motor brain dynamics for real objects versus planar images. J. Neuroimage..

[21] Oliver ZJ, Cristino F, Roberts MV, Pegna AJ, Leek EC (2018). Stereo viewing modulates three-dimensional shape processing during object recognition: A high-density ERP study. J. Exp. Psychol..

[22] Akay A, Celebi G (2009). A brain electrophysiological correlate of depth perception. J. Neurosci..

[23] Pegna JA, Darque A, Roberts MV, Leek EC (2018). Effects of stereoscopic disparity on early ERP components during classification of three-dimensional objects. J. Exp. Psychol..

[24] Liu B, Meng X, Wu G, Dang J (2013). Correlation between three-dimensional visual depth and N2 component: Evidence from event-related potential study. J. Neurosci...

[25] Roberts KL, Allen HA, Dent K, Humphreys GW (2015). Visual search in depth: The neural correlates of segmenting a display into relevant and irrelevant three-dimensional regions. J. Neuroimage..

[26] Van den Berg B, Appelbaum LG, Clark K, Lorist MM, Woldorff MG (2016). Visual search performance is predicted by both prestimulus and poststimulus electrical brain activity. J. Sci Rep..

[27] Kasai T, Morotomi T (2001). Event-related brain potentials during selective attention to depth and form in global stereopsis. J. Vis. Res..

[28] Frey J, Appriou A, Lotte F, Hachet M (2016). Classifying EEG signals during stereoscopic visualization to estimate visual comfort. J. Comput. Intell. Neurosci..

[29] Kim YJ, Lee EC (2011). EEG based comparative measurement of visual fatigue caused by 2D and 3D displays. Springer.

[30] Chen, W. Multidimensional characterization of quality of experience of stereoscopic 3D TV. Preprint at https://theses.hal.science/tel-00785987 (2012).

[31] Chen C, Li K, Wu Q, Wang H, Qian Z, Sudlow G (2013). EEG-based detection and evaluation of fatigue caused by watching 3DTV. J. Displays..

[32] Malik AS (2015). EEG based evaluation of stereoscopic 3D displays for viewer discomfort. Biomed. Eng..

[33] Murata A, Uetake A, Takasawa Y (2005). Evaluation of mental fatigue using feature parameter extracted from event-related potential. J. Int. J. Ind. Ergonom..

[34] Bader P, Henze N, Broy N, Wolf K (2016). The effect of focus cues on separation of information layers. ACM.

[35] Osmanis K (2018). Advanced multiplanar volumetric 3d display. J. Emerg. Liquid Cryst. Technol. XIII..

[36] Zhan T, Xiong J, Zou J, Wu ST (2020). Multifocal displays: Review and prospect. J. PhotoniX..

[37] Smalley D, Poon TC, Gao H, Kvavle J, Qaderi K (2018). Volumetric displays: Turning 3-D inside-out. J. Opt. Photon. News..

[38] Li Y, Zhang C, Hou C, Yao L, Zhang J, Long Z (2017). Stereoscopic processing of crossed and uncrossed disparities in the human visual cortex. J. BMC Neurosci..

[39] Jasper HH (1958). The ten twenty electrode system of the International Federation. J. Elec. Clin. Neurophysiol..

[40] Akyürek EG, Dinkelbach A, Schubö A (2010). The neural processing fate of singleton target and nontarget stimuli. J. Brain Res..

[41] Vogel EK, Luck SJ (2000). The visual N1 component as an index of a discrimination process. J. Psychophysiol..

[42] Freunberger R, Klimesch W, Doppelmayr M (2007). Visual P2 component is related to theta phase-locking. J. Neurosci. Lett..

[43] Omoto, S., Kuroiwa, Y., Otsuka, S., Baba, Y., Wang, C., Li, M., … Suzuki, Y. P1 and P2 components of human visual evoked potentials are modulated by depth perception of 3-dimensional images. *J. Clin. Neurophysiol*. **121**, 386–391 (2010). 10.1016/j.clinph.2009.12.005 20071231

[44] Haider A, Fazel-Rezai R (2017). Application of P300 event-related potential in brain-computer interface. J. Event-Relat. Potent. Evoked Potent..

[45] Salti M, Bar-haim Y, Lamy D (2012). The P3 component of the ERP reflects conscious perception, not confidence. J. Consciousness Cogn..

[46] Bianchi L, Sami S, Hillebrand A, Seri S (2010). Which physiological components are more suitable for visual ERP based brain—Computer interface ? A preliminary MEG / EEG study. Brain Topogr..

[47] Grossman T, Balakrishnan R (2006). An evaluation of depth perception on volumetric displays. ACM.

[48] Pladere T, Jankovska G, Konosonoka V, Panke K, Krumina G (2020). Impact of viewing distance on relative depth judgements for stimuli in physical space. J. Light Nature VII..

